# The revised ATS/ERS/JRS/ALAT diagnostic criteria for idiopathic pulmonary fibrosis (IPF) - practical implications

**DOI:** 10.1186/1465-9921-14-S1-S2

**Published:** 2013-04-16

**Authors:** Athol U Wells

**Affiliations:** 1 Royal Brompton and Harefield NHS Foundation Trust, London, UK

**Keywords:** Idiopathic pulmonary fibrosis, diagnosis, biopsy, histopathology, bronchoalveolar lavage, multidisciplinary

## Abstract

Idiopathic pulmonary fibrosis (IPF), the most prevalent idiopathic interstitial pneumonia, is associated with a poor prognosis. An accurate diagnosis of IPF is essential for optimal management. The recent ATS/ERS/JRS/ALAT recommendations on the diagnosis and management of IPF were developed from a systematic review of the published literature. High-resolution computed tomography (HRCT) scanning has a central role in the IPF diagnostic pathway with formal designation of criteria for an HRCT pattern of UIP. In the correct clinical context, a UIP pattern on HRCT is indicative of a definite diagnosis of IPF without the need for a surgical lung biopsy. However, although the 2011 ATS/ERS/JRS/ALAT statement is a major advance, the application of guideline recommendations by clinicians has identified limitations that need to be addressed in future statements. Key problems include: 1) the lack of management recommendations for the highly prevalent clinical scenarios of probable and possible IPF; 2) the ongoing confusion about the diagnostic role of bronchoalveolar lavage (reflecting ambiguity in the current recommendation); 3) HRCT misdiagnosis by less experienced radiologists, increasingly recognised as a major problem; and 4) the lack of integration of clinical data, including the treated course of disease, in the designation of the diagnostic likelihood of IPF.

## Introduction

Idiopathic Interstitial Pneumonias (IIPs) make up a heterogeneous group of diseases, which are collectively included in the umbrella term “Interstitial Lung Diseases (ILDs)” [1]. In 2002, the ATS/ERS multidisciplinary panel proposed a classification of IIPs that comprises clinicopathological entities such as Idiopathic Pulmonary Fibrosis (IPF), Nonspecific Interstitial Pneumonia (NSIP), Respiratory Bronchiolitis-associated Interstitial Lung Disease (RB-ILD), Cryptogenic Organising Pneumonia (COP), Acute Interstitial Pneumonia (AIP), Desquamative Interstitial Pneumonia (DIP), and lymphoid interstitial pneumonia (LIP) [1].

IPF is defined as a specific form of chronic, progressively fibrosing IIP, occurring primarily in older adults, limited to the lungs, and associated with the histopathological and/or radiologic pattern of unspecified interstitial pneumonia (UIP) [2]. IPF is the most common and pernicious ILD [3]. In addition, the histologically confirmed UIP pattern of IPF is shown to be associated with a significantly worse prognosis than other histologic patterns of chronic interstitial pneumonia [4]. The diagnosis of IPF requires an integrated multidisciplinary approach involving pulmonologists, radiologists, and pathologists. Hence, establishing an accurate diagnosis of IPF is a challenging process. This clinical review focuses on the revised diagnostic criteria (2011) for IPF and discusses the feasibility of their application in the clinical setting. Key problems that have emerged with the application of the guidelines by clinicians are discussed.

## The ATS/ERS/JRS/ALAT 2011 revised diagnostic criteria

An IPF diagnosis is based the absence of a known cause of lung fibrosis, computed tomography (CT) findings and, in cases with CT abnormalities that are not classical for IPF, the use of pathological criteria. Diagnostic uncertainties, partly due to the multiple different ways in which physicians approach IPF (e.g. the availability of appropriate lung biopsy specimens and accurate medical histories) along with the variability in the natural history of disease and in HRCT appearances; and the lack of a validated algorithm for excluding known causes of lung fibrosis all contribute to the inherent confusion surrounding this topic.

The lack of a known cause of lung fibrosis represents a key factor in the diagnostic process. Even when a surgical lung biopsy (SLB) reveals a histopathological pattern of UIP, a definitive diagnosis required the exclusion of other known causes of ILD, including collagen vascular disease, drug toxicity, and various environmental exposures (Table 1) [1,5]. In some patients, surgical lung biopsy (SLB) is unnecessary as the diagnosis of IPF is secure, based on typical clinical and HRCT features. The statement in 2000 identified four major and four minor diagnostic criteria for IPF, with a requirement for the presence of all four major and three of the four minor criteria (Table 2) [1,5]. However, all four minor criteria have significant limitations. The requirements for a slow onset of disease and a duration of at least three months do not acknowledge the fact that some IPF patients first present with an acute exacerbation. Furthermore, in patients with symptoms from pre-existing smoking-related lung damage, it can be impossible to accurately evaluate the separate symptomatic course of coexisting IPF. Finally, the presence of crackles on auscultation is a highly non-specific sign.

**Table 1:** Clinical conditions associated with usual interstitial pneumonia pattern [1]

Idopathic pulmonary fibrosis/cryptogenic fibrosing alveolitis
Collagen vascular disease
Drug toxicity
Chronic hypersensitivity pneumonitis
Asbetosis
Familial idopathic pulmonary fibrosis
Hermansky-Pudlak syndrome

**Table 2:** ATS/ERS criteria for diagnosis of idiopathic pulmonary fibrosis in absence of surgical lung biopsy (2000)^†^[1]

Major criteria
Exclusion of other known causes of ILD such as certain drug toxicities, environmental exposures, and connective tissue diseases Abnormal pulmonary function studies that include evidence of restriction (reduced VC, often with an increased FEV_1_/FVC ratio) and impaired gas exchange [increased P(A-a)O_2_, decreased PaO_2_ with rest or exercise or decreased DL_CO_] Bibasilar reticular abnormalities with minimal ground glass opacities on HRCT scans Transbronchial lung biopsy or BAL showing no features to support an alternative diagnosis

**Minor criteria**

Age >50 yr Insidious onset of otherwise unexplained dyspnoea on exertion Duration of illness >3 months Bibasilar, inspiratory crackles (dry or “Velcro”-type in quality)

Definition of abbreviations: BAL=bronchoalveolar lavage; DL_CO_=diffusing capacity of the lung for CO; HRCT=high-resolution computerised tomography; ILD=interstitial lung disease; P(A–a)O_2_=alveolar–arterial pressure difference for O_2_; VC=vital capacity.

^†^In the immunocompetent adult, the presence of all of the major diagnostic criteria as well as at least three of the four minor criteria increases the likelihood of a correct clinical diagnosis of IPF.

The updated evidence-based guidelines for diagnosis and management of IPF were devised in 2011 by the collaborative effort of the American Thoracic Society, the European Respiratory Society, the Japanese Respiratory Society, and the Latin American Thoracic Association. The main objective was to provide recommendations on the diagnosis and management of IPF, based on a thorough review of the evidence published to date, and the use of expert opinion only when the evidence base is inadequate.

The diagnostic criteria from 2000 indicate that in the correct clinical context, the finding of a UIP pattern on a high resolution computed tomography (HRCT) scan may be sufficient to diagnose IPF, without the need to perform SLB [1]. This conclusion is consolidated in the 2011 guidelines, in which HRCT has a primary role in the IPF diagnostic pathway. Imaging criteria for the diagnosis of a UIP pattern on HRCT include the presence of bilateral, predominantly subpleural, basal reticular abnormalities and the absence of additional features considered incompatible with a diagnosis of IPF. However, these HRCT features are only indicative that IPF is a possible diagnosis: a definite diagnosis requires the presence of honeycombing, a much more specific feature of a UIP pattern [2]. The HRCT features of honeycombing are, clustered cystic air space, usually of comparable diameters (3–10 mm, occasionally larger), mainly subpleural and characterised by thick well-defined walls [2]. Honeycombing on HRCT in an appropriate distribution has a positive predictive value for a histologic pattern of UIP ranging from 90-100% [^6^].

The ATS/ERS/JRS/ALAT 2011 guidelines clearly state the precise HRCT features that meet the criteria for ‘‘UIP’’, ‘‘possible UIP’’ and ‘‘inconsistent with UIP’’ patterns (Tables 3 &4) [2]. In the appropriate clinical setting, satisfaction of HRCT criteria for a pattern of UIP obviates a diagnostic SLB. In patients demonstrating features that meet the criteria for ‘‘possible UIP’’ and/or ‘‘inconsistent with UIP’’ patterns on HRCT images, a SLB should be considered. Specific combinations of HRCT and histopathology patterns are also provided in the statement with delineation of the likelihood of IPF as definite, probable or possible [2].

**Table 3:** High-resolution computed tomography criteria for uip pattern [2]

UIP Pattern (All Four Features)	Possible UIP Pattern (All Three Features)	Inconsistent with UIP Pattern (any of the Seven Features)
■ Subpleural, basal predominance	■ Subpleural, basal predominance	■ Upper or mid-lung predominance
■ Reticular abnormality	■ Reticular abnormality	■ Peribronchovascular predominance
■ Honeycombing with or without traction bronchiectasis	■ Absence of features listed as inconsistent with UIP pattern (*see* third column)	■ Extensive ground glass abnormality (extent >reticular abnormality)
■ Absence of features listed as inconsistent with UP		■ Profuse micronodules (bilateral, predominantly upper lobes)
		■ Discrete cysts (multiple, bilateral, away from areas of honeycombing)
		■ Diffuse mosaic attenuation/air-trapping (bilateral in three or more lobes)
		■ Consolidation in bronchopulmonary segment(s)/lobe(s)

**Table 4:** Combination of high-resolution computed tomography and surgical lung biopsy for the diagnosis of ipf (requires multidisciplinary discussion) [2]

HRCT Pattern	Surgical Lung Biopsy Pattern (When Performed)		Diagnosis of IPF?*
**UIP**	**UIP**	**}**	**YES**
	**Probable UIP**		
	**Possible IUP**		
	**Nonclassifiable fibrosis^†^**		
	Not UIP		No
**Possible UIP**	**UIP**	**}**	**YES**
	**Probable UIP**		
	Possible UIP	**}**	Probable^‡^
	Nonclassifiable fibrosis		
	Not UIP		No
Inconsistent with UIP	UIP		Possible^‡^
	Probable UIP	**}**	No
	Possible UIP		
	Nonclassifiable fibrosis		
	Not UIP		

* The accuracy of the diagnosis of IPF increases with multidisciplinary discussion (MDD). This is particularly relevant in cases in which the radiologic and histopathologic patterns are discordant (e.g., HRCT is inconsistent with UIP and histopathology is UIP). There are data to suggest that the accuracy of diagnosis is improved with MDD among interstitial lung disease experts compared to clinician-specialists in the community setting; timely referral to interstitial lung disease experts is encouraged.

†Nonclassifiable fibrosis: Some biopsies may reveal a pattern of fibrosis that does not meet the above criteria for UIP pattern and the other idiopathic interstitial pneumonias. These biopsies may be termed ‘‘nonclassifiable fibrosis.’’

Multidisciplinary discussion should include discussions of the potential for sampling error and a re-evaluation of adequacy of technique of HRCT. NOTE: In cases with an ‘‘inconsistent with UIP’’ HRCT pattern and a ‘‘UIP’’ surgical lung biopsy pattern, the possibility of a diagnosis of IPF still exists and clarification by MDD among interstitial lung disease experts is indicated.

A multidisciplinary, dynamic approach, with the input of clinicians, radiologists, and pathologists has been shown to improve diagnostic accuracy [7,8] and is strongly recommended. In the ATS/ERS/JRS/ALAT statement, no attempt is made to define minimum standards required for the process of multidisciplinary evaluation.

## Management guidance in relation to diagnostic likelihood of idiopathic pulmonary fibrosis

A key problem in applying the updated guidelines on management is related to the designation of IPF as “definite”, “probable” or “possible” (Tables 3 &4) [2]. On one level, this aspect of the guidelines is a major advance as there has been no previous definition of probable and possible IPF, even though both are frequently encountered in clinical practice. However, it must be acknowledged that the recommendations provided in the new evidence-based guidelines are focused on patients with a definite diagnosis of IPF and are often difficult to apply when the diagnosis of IPF is less certain. When a realistic differential diagnosis exists (usually chronic hypersensitivity pneumonitis [HP] or fibrotic non-specific interstitial pneumonia [NSIP]), the less interventional approach recommended in IPF may lead to the under-treatment of disorders other than IPF, with major adverse consequences. The appropriate recommendation is also made that a SLB should be performed when the diagnosis of IPF is uncertain. However, a significant minority (perhaps a large minority) of patients with suspected IPF cannot undergo a SLB procedure, either because of contraindications (comorbid conditions, disease severity, advanced age), or because the patient declines the procedure. Thus, IPF patients presenting with non-diagnostic scans must undergo a SLB or be disenfranchised by current diagnostic criteria. This scenario is especially a difficulty in elderly patients who are unwilling or unable to undergo a SLB and are therefore debarred from receiving novel therapies which are licensed for use in definite IPF. The most prevalent problem is the IPF patient without honeycombing on HRCT but with a predominantly basal and sub-pleural distribution of reticular abnormalities that is typical of IPF, which is also seen in chronic HP and fibrotic NSIP. There is an urgent need to repeat the study of Fell et al. [9], in which it was shown that in patients aged over 65 with reticular abnormalities (as described above) but no honeycombing, the diagnostic likelihood of IPF exceeded 95%. Confirmation of this finding in another large cohort would allow the current HRCT criteria for a diagnosis of definite IPF to be expanded.

## The best diagnostic use of bronchoalveolar lavage

The value of bronchoalveolar lavage (BAL) in the diagnosis of IPF is contentious. In patients with suspected IPF, the most important application of BAL is to increase the index of suspicion for alternative disorders, including HP and NSIP, by the demonstration of a lymphocytosis >30%. The ATS/ERS/JRS/ALAT 2011 guidelines make a weak negative recommendation that BAL cellular analysis should not be performed in the diagnostic evaluation of IPF in the majority of patients, but may be appropriate in a minority of patients [2].

However, feedback from European practitioners indicates that this recommendation has caused a good deal of confusion as it can be applied to either of two very separate scenarios. At the time of BAL, a HRCT scan has almost always been performed. If HRCT features are classical for IPF, and the clinical context is correct, the patient does not have “suspected IPF” but “definite IPF”. If the HRCT features are not classical for IPF, but IPF is suspected, the diagnostic overlap includes IPF/NSIP, IPF/HP, IPF/sarcoidosis and IPF/unclassifiable. It is not helpful to combine these two scenarios in a single recommendation. Two independent statements are required. Thus, some clinicians take the recommendation to apply to patients, with clinical and HRCT features typical of IPF on HRCT (“definite IPF”). Others assume that the recommendation does indeed apply to “suspected IPF”, in which, by definition, the likelihood of IPF is probable or possible but realistic differential diagnoses exist, usually of HP or NSIP. These two scenarios differ radically.

Regarding the first scenario (typical clinical and HRCT features of IPF), it is likely that most clinicians would agree that an additional diagnostic test is not required. As occasional patients have uncertain exposures, which might or might not be relevant to a diagnosis of HP, a weak negative recommendation for the use of BAL, reserved for those patients, seems appropriate. However, even this apparently logical conclusion can be questioned. In a study by Ohshimo et al., of the diagnostic significance of a BAL lymphocytosis when the clinical and HRCT presentation is typical of IPF [10], a BAL lymphocytosis of >30% was present in six out of 74 patients and in all six cases an alternative diagnosis to IPF was made. The change in diagnostic perception was validated by surgical biopsy in two cases, and by the subsequent outcome (which was not typical of IPF) in four cases. This finding, applying to a handful of patients, needs to be reproduced in multicenter studies with a larger cohort of patients before major changes in the recommendation are considered.

By contrast, when the diagnosis of IPF is “suspected” and, thus, by definition, uncertain, most European clinicians would favour the performance of BAL, with findings expected to radically alter the likelihood of alternative diagnoses. This view was endorsed by formal voting in a large European-wide meeting of clinicians with expertise in IPF and flies directly in the face of the ATS/ERS/JRS/ALAT 2011 recommendation. This is likely to reflect the fact that the ATS/ERS/JRS/ALAT recommendation covered two such dissimilar clinical scenarios with a single recommendation, although cultural differences between Europe and the USA in the performance of BAL might also have played an important role. However, irrespective of the source of confusion, there is a perceived need for separate recommendations to address the two distinct clinical scenarios, as in the British Thoracic Society (BTS) guidelines on the diagnosis and management of interstitial lung disease. In the BTS guidelines, the diagnostic use of BAL was reserved for patients in whom the diagnosis is uncertain after clinical assessment and HRCT scanning, with the case by case exclusion of patients with severe disease or because a biopsy will ultimately be performed [11]. It can be argued that a similar statement is now required for the specific diagnosis of IPF, with the two key clinical scenarios clearly distinguished.

## Guideline recommendations require expertise that is not always available

Some radiologists continue to perform sub-optimal CT scans, using historical thick section protocols (rather than high resolution CT), a practice that needs to be eradicated in the diagnostic evaluation of interstitial lung disease. This issue aside, expert HRCT (and also pathological) interpretation is central to the diagnosis of IPF and requires the identification of honeycombing. In this regard, there are two issues of major concern: the distinction between honeycombing and traction bronchiectasis, and the distinction between honeycombing and the admixture of emphysema and fibrosis. Both distinctions can be difficult, even for an experienced observer. The accuracy of a trained observer in discriminating between ILDs appears to be around 80-90% [12-14]. However, less experienced observers are substantially less accurate In the Europe-wide IPF meeting mentioned previously, it was evident, on formal voting, that a clear majority of clinicians found the HRCT diagnosis of IPF to be reliable in less than a third of practicing radiologists. Furthermore, a substantial majority of clinicians stated that IPF was over-diagnosed more often than it was under-diagnosed. This impression is consistent with findings in a retrospective review of 39 patients with diffuse parenchymal lung disease [8] there was significant disagreement in the diagnosis between community and academic practitioners, with community physicians and radiologists more likely to over-diagnose IPF.

The under-diagnosis of IPF, albeit a less prevalent problem, is also important as a confident diagnosis of IPF is made by HRCT evaluation in, at most, two thirds of IPF patients [2,15]. In at least one third of cases, IPF cannot be diagnosed from HRCT appearances. The distinction between typical and atypical HRCT appearances for a diagnosis of IPF is a major source of inter-observer variation among less experienced observers. The 2011 ATS-ERS recommendations may prove to be a major spur to better standardisation of radiological expertise by means of multidisciplinary diagnostic meetings, but do not directly address the major problem of HRCT misdiagnosis.

Clearly, training in applying guideline recommendations in the diagnosis of ILD/IPF should be directed not only to radiologists but also to pathologists and clinicians. Inter-obsever variation is a significant problem in histopathologic diagnosis. Multidisciplinary diagnosis is a relatively recent phenomenon in interstitial lung disease and there is a need for clinician training in integrating and reconciling data to distil a final accurate clinical/radiological/histologic diagnosis.

## The integration of observed disease behaviour in the diagnosis of IPF

The principle of integrating HRCT and histopathologic data in assigning a diagnostic likelihood of IPF is an admirable feature of the 2011 ATS/ERS/JRS/ALAT statement, which may well be helpful in the majority of cases and sets an important precedent for future statements. However, a problem arises in an important subgroup of patients. Sometimes, HRCT appearances are apparently incompatible with IPF but the treated disease course is classical for IPF, with inexorable progression despite therapy. Often, in this setting, a SLB reveals histologic abnormalities that are absolutely typical of IPF. Using the schema advanced in the ATS/ERS/JRS/ALAT 2011 statement, such patients cannot be diagnosed as having definite or probable IPF but must be classified as having possible IPF. This is a deeply unsatisfactory state of affairs. A SLB is often performed in these cases because the treated course of disease is typical of IPF [2] and atypical for the alternative diagnosis suggested by HRCT. Plainly, in such patients, IPF is, at the very least, a possible diagnosis and, sometimes, a probable diagnosis, before SLB is performed. Does the demonstration of a classic UIP pattern on biopsy really have no impact on the diagnostic likelihood of IPF in this scenario? Is the diagnosis of IPF truly no more than a possible diagnosis, once the biopsy findings are integrated? This unhappy conclusion is seriously unhelpful for patients, relatives and clinicians who see the performance of SLB as a means of reducing diagnostic uncertainty. Perhaps patients with HRCT appearances “not compatible” with IPF should not be included in trials of new therapies in IPF, but it would be helpful if the next major statement on the diagnosis of IPF contains an explicit acknowledgement that a definite multidisciplinary diagnosis of IPF can sometimes be made, based on biopsy findings and a typical treated course, even when the HRCT features are apparently “incompatible” with a diagnosis of IPF.

A deficiency common to all guideline statements is the absence of pragmatic guidance on the level of risk of SLB in individual patients. A clearer separation between patients who can undergo a SLB with an acceptably low risk and those in whom SLB should be avoided would be helpful to clinicians. The presence of major co-morbidity is an obvious contraindication to SLB but the level of severity of IPF associated with a significant increase in post-operative mortality has not been sufficiently addressed. It is acknowledged that in the absence of definitive data, broad statements based on indirect evidence might be required. However, guidance on this point, even based solely on expert opinion, would be extremely useful as a means of facilitating patient participation in decisions based on “risk-benefit” thinking.

## Conclusions

The evidence-based ATS/ERS/JRS/ALAT 2011 guideline recommendations have assisted clinicians in making a diagnosis and subsequent treatment decisions for the clinical management of IPF. The guidelines are a major improvement on the previous statements of the committee that were published in the early 2000s, with an impressive review of the literature and a disciplined approach to a previously undisciplined field. However, in common with all such statements, guidance mainly focuses on definite IPF, most often diagnosed from typical HRCT appearances. The definition of probable and possible IPF is also an advance, but the failure to provide “intention to manage” guidance on these highly prevalent clinical scenarios is a major limitation.

Other aspects of the diagnostic guidelines would merit review, based on problems that have become increasingly apparent as recommendations have been applied. The BAL recommendation has caused confusion due to the use of a single recommendation to cover two radically different clinical scenarios. The over-diagnosis of IPF on HRCT appears to be widespread, with urgent work needed on the standardisation of HRCT diagnosis by expert practitioners. The integration of HRCT and histologic data in the assigning of diagnostic likelihood is more often helpful than not, but does not meet the needs of some patients in whom a multidisciplinary diagnosis of definite IPF should be made, based on the treated course of disease and typical biopsy appearances, even when HRCT appearances are non-diagnostic or suggestive of an alternative diagnosis. As with any other disease, these issues highlight the continued need for updating the guidelines, including diagnostic and therapeutic aspects, based upon the limitations that become evident as recommendations are applied.

Pending revision by expert groups, awareness of the flaws in the current IPF guidelines should alert clinicians to the need to reject or modify guideline recommendations in individual patients, provided that these decisions can be defended at multidisciplinary review.

## Competing interests

Athol U. Wells has received consultancy fees from Actelion, Centricorps, Gilead and Intermune and has also received fees for participating as a speaker in symposia sponsored by Actelion and Intermune.

## Acknowledgements

The author thanks C. Trenam, I. Mandic and M. Smith of IntraMed Communications for editorial assistance in the preparation of the manuscript. Development of this article was supported by InterMune AG.

## Declarations

This article has been published as part of *Respiratory Research* Volume 14 Supplement 1, 2013:IPF in 2011 – Key updates on guidelines and therapeutics. The full contents of the supplement are available online at http://respiratory-research.com/supplements/14/S1. Publication of this supplement was supported by IntraMed Communications with funding from InterMune, AG. InterMune is the manufacturer of pirfenidone, a product mentioned in this article. The supplement originates from presentations given at the “AIR Event: Advancing IPF Research. Working together to translate IPF research into practice” held in Berlin in November 2011. The publication was proposed by IntraMed Communications and developed in consultation with the journal. All articles in the supplement have undergone the journal’s standard peer review process.
